# Association between the oxidative balance score and testosterone deficiency in males: a cross-sectional study

**DOI:** 10.3389/fnut.2025.1577823

**Published:** 2025-07-23

**Authors:** Ze Zhuge, Kaihui Zheng, Xiaojun Ji, Xiaobo Wang, Xuena Zhang

**Affiliations:** Wenzhou Central Hospital, Wenzhou, China

**Keywords:** testosterone deficiency, oxidative balance score, lifestyle, dietary, NHANES

## Abstract

**Background:**

Testosterone deficiency is a prevalent condition among males and warrants greater attention. The balance between oxidative and antioxidant capacity plays a critical role in testosterone deficiency.

**Methods:**

This study conducted a secondary analysis using data from three cycles of the National Health and Nutrition Examination Survey (2011–2016). The oxidative balance score (OBS) and testosterone levels were derived from interview data and laboratory measurements. Weighted logistic regression was employed to explore the relationship between OBS and testosterone deficiency. Subgroup analyses, sensitivity analyses, and *p*-value for interaction were also performed.

**Results:**

The analysis encompassed a cohort of 3,578 participants who met the eligibility criteria. The prevalence of testosterone deficiency among participants was 23.69%. OBS was inversely correlated with testosterone deficiency, with each unit increase in OBS associated with a 3% reduction in the risk of testosterone deficiency (OR, 0.97; 95% CI, 0.95 to 0.99). The highest OBS group exhibited a 38% lower risk of testosterone deficiency (OR, 0.62; 95% CI, 0.40 to 0.96) Compared to the lowest OBS group. Furthermore, lifestyle OBS was also negatively correlated with testosterone deficiency. In, subgroup analyses, the inverse association between OBS and testosterone deficiency was most pronounced in subgroups characterized by educational background beyond high school, PIR > 3, and absence of hypertension or diabetes.

**Conclusion:**

Higher OBS was inversely correlated with testosterone deficiency in males. This study underscores the importance of comprehensive antioxidant approaches, particularly lifestyle OBS, in male testosterone deficiency.

## 1 Introduction

The health concerns of males should not be overlooked or marginalized. In the adult male population of the United States, the prevalence of low testosterone is approximately 5.6%. With the progression of societal aging, the socio-medical burden associated with testosterone deficiency is further exacerbated (1). Testosterone plays a pivotal role in the maintenance of male characteristics and the development of the musculoskeletal system (2). Despite the development of certain pharmacological interventions for treatment, these measures are unable to fundamentally alter the current situation (3, 4). Low testosterone levels are associated with various diseases, such as osteoarthritis, cardiovascular disease, and pulmonary health (5–7). Consequently, addressing the current prevalence of male testosterone deficiency and developing evidence-based intervention strategies carries epoch-making significance for alleviating both societal and healthcare burdens.

Previous studies on the effect of diet and lifestyle on testosterone levels have shortcomings or limitations, which may prevent people (men) from doing a good job of intervention according to diet and lifestyle. For example, the oxidative properties of foods and medications encountered in daily life can significantly influence testosterone levels. Sodium fluoride, a substance widely present in drinking water and food, can influence testosterone production by altering oxidative stress levels in the testes (8). Vitamin C, acting as an antioxidant, can reverse the decreased testosterone levels induced by lead exposure (9). Low levels of vitamin B12 are associated with an increased risk of testosterone deficiency (10). The antioxidant properties of curcumin also significantly influence testosterone levels in the body (11). However, human dietary intake inherently comprises complex combinations of nutrients rather than isolated compounds. Of particular importance is the recognition that environmental xenobiotics can exert testosterone-disrupting effects through oxidative mechanisms. Glyphosate has been demonstrated to elevate oxidative levels (12), and exposure to glyphosate in the environment has been shown to reduce levels of sex hormones, including testosterone (13). Male non-smokers exhibit significantly higher testosterone levels compared to the median testosterone level of the general male population (14). Multiple lifestyle factors should be systematically incorporated into the research framework. Therefore, modulating the body’s oxidative balance may offer promising prospects for individuals with low testosterone levels. Current research has primarily concentrated on the effects of modifying individual oxidative factors on testosterone levels, overlooking the broader role of overall oxidative status in testosterone regulation. However, there is an Oxidative Balance Score (OBS), that provides a comprehensive assessment of systemic redox homeostasis, offering distinct advantages over isolated examinations of dietary or lifestyle factors. Its principal strength lies in the holistic evaluation of human physiology, which integrates both pro-oxidant and antioxidant exposures, accounts for biological interactions between nutritional and behavioral components, and reflects the organism’s net oxidative stress burden. It provides a basis for future research and the formulation of relevant preventive healthcare measures.

A higher OBS indicates a stronger antioxidative capacity and it has emerged in recent years as a significant metric for assessing the balance between oxidative and antioxidative capacities within the body. Currently, OBS has been demonstrated to be associated with conditions such as sleep disorders and pulmonary diseases, showcasing its considerable potential for clinical application (15, 16). Previous studies have highlighted the intricate relationship between dietary/lifestyle factors and low testosterone levels, yet there is a notable lack of research focusing on the role of antioxidative status in populations with low testosterone. Consequently, the potential association between overall oxidative-antioxidative status and low testosterone represents a promising area of investigation. We hypothesize that there exists an inverse correlation between OBS and the risk of testosterone deficiency. Understanding this relationship is crucial for the management of male testosterone deficiency. We anticipate that integrated antioxidant approaches combining dietary and lifestyle interventions will provide effective prevention and clinical guidance for males with testosterone deficiency.

## 2 Materials and methods

### 2.1 Study population and design

The National Health and Nutrition Examination Survey (NHANES) provides comprehensive data on disease and nutritional status across a broad segment of the U. S. population, encompassing physical examinations, laboratory tests, and questionnaire surveys, making its data highly representative. As such, it serves as a valuable resource for generating evidence and references to inform public health policies aimed at disease prevention.

**Figure 1:**
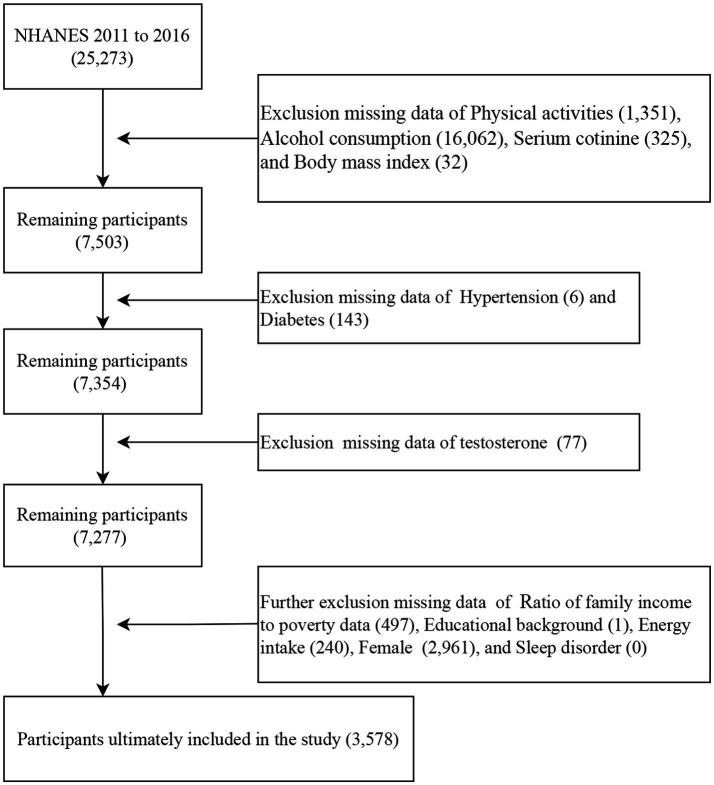
Population selection.

This study analyzed data from three NHANES survey cycles (2011–2012, 2013–2014, and 2015–2016). These cycles were selected because they contain complete data necessary for calculating the OBS as well as testosterone measurements. First, 25,273 participants with complete dietary OBS data were included. Second, participants with missing data on physical activities (1,351), alcohol consumption (16,062), serum cotinine (352), and body mass index (BMI) (17) were excluded. Third, participants with missing data on hypertension (6) and diabetes (143) were excluded. Then, participants with missing data on testosterone level (77) were excluded. In addition, participants with missing data on PIR (497), educational background (1), energy intake (240), females (2,961), and sleep disorder (0) were also excluded. The analysis for this study ultimately included a total of 3,578 participants (Figure 1).

### 2.2 Ethics statement

This study analyzed de-identified data from NHANES, which obtained the National Center for Health Statistics institutional review board approval and participant consent during primary data collection (Protocol #2011–17). The use of public data was exempt from further review per institutional guidelines.

### 2.3 Exposure definition

The OBS comprises a total of 5 pro-oxidants and 15 antioxidants. OBS was further divided into dietary OBS and lifestyle OBS. First, information required to calculate the OBS for each participant was collected. To ensure the accuracy of dietary intake, the final intake values were calculated based on the results of the average of the two 24-h interviews. Second, lifestyle OBS includes alcohol consumption, serum cotinine, BMI, and physical activity. Average alcohol consumption over the past 12 months was defined as alcohol intake. Given the increased exposure to secondhand smoke, this study measured smoking utilized serum cotinine levels, a metabolite of cigarette smoke. Physical activity was defined using the formula from this literature (18).

In this study, each component of the Oxidative Balance Score (OBS) was assigned a score based on its tertile distribution. Specifically, antioxidant components were scored as high (2), medium (1), and low (0) levels, respectively, while pro-oxidant components were scored inversely. The aggregate sum of their addition was denoted as OBS (Table 1). Additionally, the lifestyle/dietary OBS were also calculated separately. The scoring system is designed to quantitatively reflect systemic oxidative stress homeostasis.

**Table 1:** Composition of OBS in males.

OBS components	Property	Male		
		0	1	2
Dietary OBS components				
Dietary fiber (g/d)	A	<14.05	14.05–22.30	≥22.30
Carotene (RE/d)	A	<604.50	604.50–1,931.50	≥1,931.50
Riboflavin (mg/d)	A	<1.90	1.90–2.66	≥2.66
Niacin (mg/d)	A	<24.88	24.88–33.87	≥33.87
Vitamin B6 (mg/d)	A	<1.86	1.86–2.74	≥2.74
Total folate (mcg/d)	A	<337.50	337.50–502.00	≥502.00
Vitamin B12 (mcg/d)	A	<3.79	3.79–6.19	≥6.19
Vitamin C (mg/d)	A	<38.78	38.78–96.70	≥96.70
Vitamin E (ATE) (mg/d)	A	<6.83	6.83–10.60	≥10.60
Calcium (mg/d)	A	<807.00	807.00–1,209.00	≥1,209.00
Magnesium (mg/d)	A	<276.50	276.50–377.50	≥377.50
Zinc (mg/d)	A	<10.17	10.17–14.37	≥14.37
Copper (mg/d)	A	<1.05	1.05–1.49	≥1.49
Selenium (mcg/d)	A	<107.75	107.75–148.60	≥148.60
Total fat (g/d)	P	≥103.49	73.43–103.49	<73.59
Iron (mg/d)	P	≥17.53	12.64–17.53	<12.64
Lifestyle OBS components				
Physical activity (MET-minute/week)	A	<1,680.00	1,680.00-5,520.00	≥5,520.00
Alcohol (g/d)	P	≥3.00	2.00–3.00	<2.00
Body mass index (kg/m2)	P	≥29.60	25.50–29.60	<25.50
Cotinine (ng/mL)	P	≥1.43	0.02–1.43	<0.02

A stood in for the antioxidant, *p* for the pro-oxidant. RE for the retinal equivalent, ATE for the alpha-tocopherol equivalent, and MET for the metabolic equivalent.

### 2.4 Outcome definitions

Specimens were collected by professionally trained personnel. Total testosterone levels in serum were measured using isotope dilution liquid chromatography tandem mass spectrometry (ID-LC–MS/MS). A testosterone level below 300 ng/dL is defined as testosterone deficiency (19).

### 2.5 Covariates definitions

There were some covariates considered in this study, including age, race/ethnicity, poverty-to-income ratio (PIR), educational background, total energy intake, diabetes status, and hypertension status. Extreme dietary intake will be excluded (<800 or >4,200 kcal day^−1^ for males) (20). Given the well-established association between hypertension, diabetes, and testosterone deficiency in males (21, 22). Therefore, hypertension and diabetes were incorporated as covariates in this study. The presence of chronic diseases was determined based on self-reported hypertension or diabetes. Numerous studies have reported a robust association between sleep disorder and testosterone deficiency (23, 24). Therefore, we incorporated sleep disorders as a covariate in the study, defined based on self-reported sleep disorder questionnaires (25).

### 2.6 Statistical analysis

The data in this study were available directly from the official NHANES website. During the data analysis process, the data were weighted to ensure representativeness. Normality tests were conducted for continuous variables, and non-normally distributed continuous variables were expressed using medians. Categorical variables were presented as unweighted frequencies (weighted percentages).

When examining the baseline characteristics across OBS quartiles, categorical variables were analyzed using the Rao-Scott chi-square test, while non-normally distributed continuous variables were assessed using the Kruskal-Wallis test. Weighted logistic regression was employed to investigate the potential relationship between OBS and low testosterone in males.

This study further stratified OBS into lifestyle OBS and dietary OBS to separately examine their associations with low testosterone in males. Additionally, subgroup analyses were performed to identify high-risk populations. Finally, sensitivity analyses were also included in this study to ensure the robustness of the findings. All analyses in this study were conducted using R (version 4.2.2), and a two-sided *p*-value of less than 0.05 was considered statistically significant.

## 3 Results

### 3.1 Baseline characteristics of participants stratified by OBS quartiles

A total of 3,578 male participants with a median age of 42 years were comprised in this study (Table 2). The prevalence of testosterone deficiency among participants was 23.69%. The majority of the participants were Non-Hispanic White. Participants in the highest OBS group, compared to those in the lowest OBS group, were more likely to have an educational background above high school, a PIR greater than 3, a higher energy intake, and no diabetes (all *p* values less than 0.05).

**Table 2:** Baseline characteristics for OBS quartiles.

		Q1 (<14)	Q2 (14–19)	Q3 (20–24)	Q4 (> = 25)	*p*-value
	*N* = 3,578	*N* = 919	*N* = 976	*N* = 768	*N* = 915	
Age (year)	42 [29; 57]	41 [28; 55]	43 [28; 57]	43 [29; 56]	43 [31; 58]	0.927
Race/Ethnicity						<0.001
Mexican American	476 (8.64%)	106 (8.30%)	135 (9.44%)	110 (8.86%)	125 (8.03%)	
Other Hispanic	320 (5.10%)	90 (5.53%)	77 (5.22%)	77 (5.31%)	76 (4.53%)	
Non-Hispanic White	1,564 (69.85%)	379 (65.65%)	417 (68.24%)	344 (70.45%)	424 (73.95%)	
Non-Hispanic Black	685 (8.37%)	258 (14.09%)	198 (9.11%)	111 (6.74%)	118 (4.61%)	
Other race	533 (8.05%)	86 (6.43%)	149 (7.99%)	126 (8.65%)	172 (8.87%)	
Educational background						<0.001
<High school	628 (11.20%)	211 (15.03%)	196 (14.39%)	127 (10.74%)	94 (5.85%)	
>High school	2,139 (68.34%)	448 (57.60%)	571 (64.14%)	467 (67.88%)	653 (80.37%)	
High school/general educational development	811 (20.46%)	260 (27.36%)	209 (21.47%)	174 (21.38%)	168 (13.77%)	
Energy intake	2,357 [1,875; 2,853]	1,793 [1,472; 2,143]	2,159 [1,775; 2,580]	2,492 [2,132; 2,910]	2,834 [2,441; 3,243]	<0.001
Poverty-to-income ratio						<0.001
<1	674 (12.39%)	224 (18.04%)	188 (12.38%)	125 (9.43%)	137 (10.29%)	
1–3	1,375 (31.89%)	390 (36.66%)	409 (35.89%)	285 (32.88%)	291 (24.10%)	
>3	1,529 (55.72%)	305 (45.30%)	379 (51.73%)	358 (57.70%)	487 (65.60%)	
Hypertension						0.229
Yes	1,085 (29.37%)	313 (31.43%)	297 (31.63%)	227 (28.34%)	248 (26.56%)	
No	2,493 (70.63%)	606 (68.58%)	679 (68.37%)	541 (71.66%)	667 (73.44%)	
Diabetes						0.003
Yes	351 (7.79%)	99 (8.48%)	102 (9.42%)	87 (10.27%)	63 (4.05%)	
No	3,227 (92.21%)	820 (91.52%)	874 (90.58%)	681 (89.73%)	852 (95.95%)	
Testosterone deficiency						0.673
Yes	846 (23.69%)	227 (24.65%)	240 (24.09%)	183 (24.86%)	196 (21.80%)	
No	2,732 (76.31%)	692 (75.35%)	736 (75.91%)	585 (75.14%)	719 (78.20%)	
Sleep disorder						0.803
Yes	741 (24.57%)	202 (24.24%)	192 (23.23%)	152 (25.09%)	195 (25.64%)	
No	2,837 (75.43%)	717 (75.76%)	784 (76.77%)	616 (74.91%)	720 (74.36%)	

OBS, oxidative balance score; Q1, the lowest OBS group; Q2, the lower OBS group; Q3, the higher OBS group. Q4, the highest OBS group.

### 3.2 Baseline characteristics for the presence or absence of testosterone deficiency

Compared to males without testosterone deficiency, those with testosterone deficiency were older, more likely to suffer from diabetes, hypertension, and sleep disorder, and also exhibited lower lifestyle OBS (all *p*-values < 0.05; Table 3).

**Table 3:** Baseline characteristics of participants stratified by the presence or absence of testosterone deficiency.

		No Testosterone deficiency	Testosterone deficiency	*p*-value
	*N* = 3,578	*N* = 2,732	*N* = 846	
Age (year)	42 [29; 57]	41 [27; 57]	46 [33; 59]	<0.001
Race/Ethnicity				0.246
Mexican American	476 (8.64%)	349 (8.45%)	127 (9.23%)	
Other Hispanic	320 (5.10%)	242 (5.18%)	78 (4.86%)	
Non-Hispanic White	1,564 (69.85%)	1,186 (69.28%)	378 (71.68%)	
Non-Hispanic Black	685 (8.37%)	542 (8.89%)	143 (6.67%)	
Other race	533 (8.05%)	413 (8.20%)	120 (7.56%)	
Educational background				0.635
<Highschool	628 (11.2%)	476 (11.13%)	152 (11.44%)	
>Highschool	2,139 (68.34%)	1,632 (68.00%)	507 (69.44%)	
Highschool/General educational development	811 (20.46%)	624 (20.88%)	187 (19.12%)	
Poverty-to-income ratio				0.246
<1	674 (12.39%)	530 (12.70%)	144 (11.37%)	
1–3	1,375 (31.89%)	1,050 (32.67%)	325 (29.38%)	
>3	1,529 (55.72%)	1,152 (54.63%)	377 (59.25%)	
Hypertension				<0.001
Yes	1,085 (29.37%)	754 (26.80%)	331 (37.64%)	
No	2,493 (70.63%)	1,978 (73.20%)	515 (62.36%)	
Diabetes				<0.001
Yes	351 (7.79%)	204 (6.15%)	147 (13.04%)	
No	3,227 (92.21%)	2,528 (93.85%)	699 (86.96%)	
Sleep disorder				<0.001
Yes	741 (24.57%)	522 (22.77%)	219 (30.36%)	
No	2,837 (75.43%)	2,210 (77.23%)	627 (69.64%)	
Energy intake	2,357 [1,874; 2,853]	2,333 [1,864; 2,854]	2,410 [1,932; 2,852]	0.600
OBS	20 [14; 25]	20 [14; 25]	19 [14; 25]	0.054
Lifestyle OBS	4 [3; 5]	4 [3; 5]	3 [2; 4 L]	<0.001
Dietary OBS	16 [10; 21]	16 [10; 21]	16 [10; 22]	0.780

### 3.3 The relationship between OBS and testosterone deficiency

**Figure 2:**
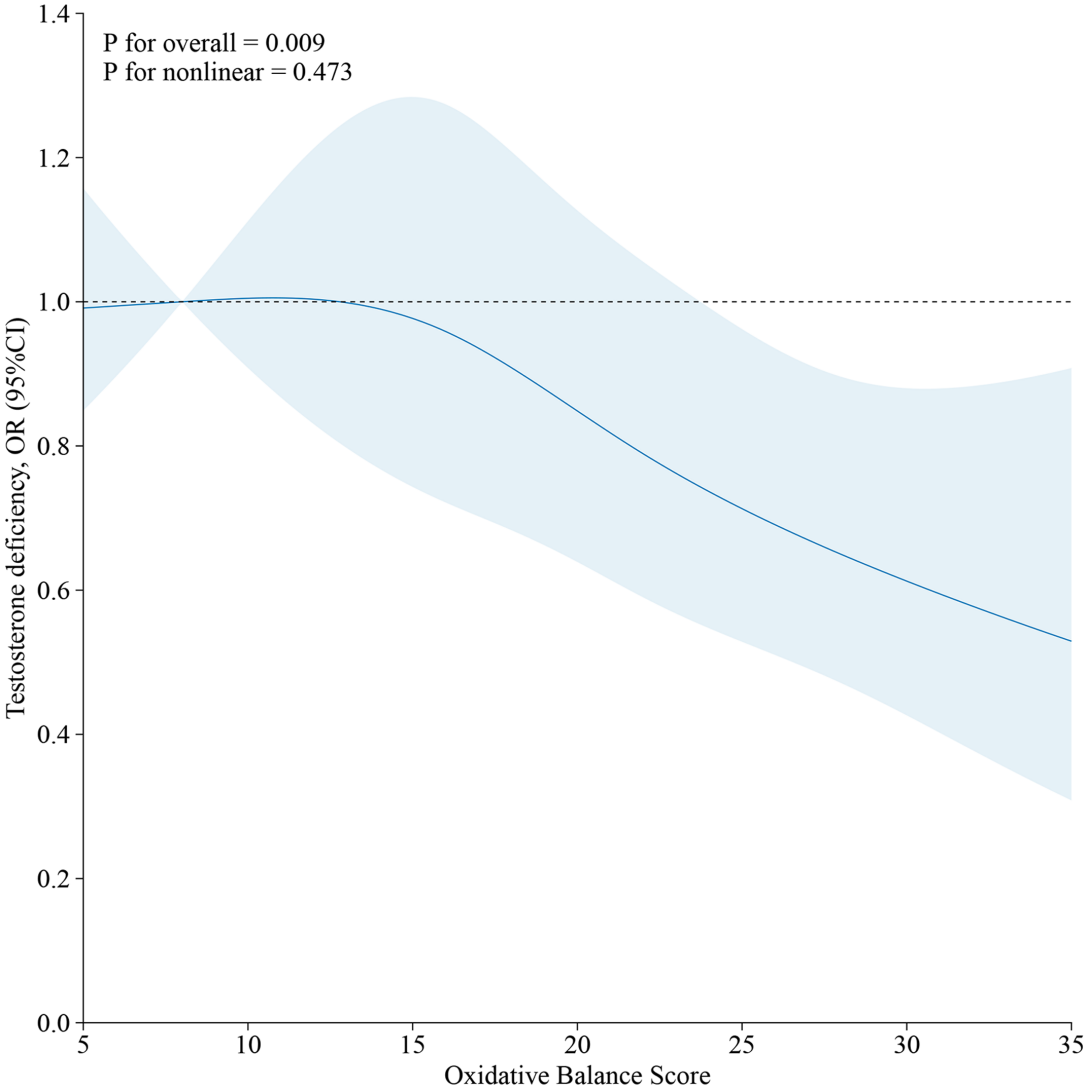
The restricted cubic spline (RCS) of the relationship between OBS and testosterone deficiency in males.

As demonstrated in Table 4, no significant association was observed between OBS and testosterone deficiency in the unadjusted Model 1. However, a significant inverse correlation between OBS and testosterone deficiency emerged (OR, 0.97; 95% CI, 0.95 to 0.99) after adjusting for all covariates. Furthermore, when using the lowest OBS group as the reference, the highest OBS group demonstrated a 38% lower risk of testosterone deficiency (OR, 0.62; 95% CI, 0.40 to 0.96), and the *p* for trend was less than 0.05. The restricted cubic spline (RCS) plot revealed a nonlinear association (p for nonlinear = 0.473) between OBS and testosterone deficiency in males (Figure 2).

**Table 4:** The relationship between OBS and testosterone deficiency.

Exposure	Testosterone deficiency, OR (95% CI)					
	Model 1	*p*-value	Model 2	*p*-value	Model 3	*p*-value
OBS (Continue)	0.99 (0.97 to 1.01)	0.234	0.99 (0.97 to 1.00)	0.125	0.97 (0.95 to 0.99)	0.009
OBS quartiles						
Q1 (<14)	Reference		Reference		Reference	
Q2 (14 to 20)	0.97 (0.74 to 1.27)	0.822	0.94 (0.71 to 1.24)	0.640	0.84 (0.63 to 1.12)	0.222
Q3 (21 to 24)	1.01 (0.71 to 1.44)	0.950	0.98 (0.68 to 1.39)	0.888	0.78 (0.53 to 1.16)	0.215
Q (> = 25)	0.85 (0.60 to 1.20)	0.352	0.81 (0.57 to 1.14)	0.217	0.62 (0.40 to 0.96)	0.031
*p* for trend		0.381		0.239		0.033

OR, odds ratio; CI, confidence interval; OBS, oxidative balance score; Q1, the lowest OBS group; Q2, the lower OBS group; Q3, the higher OBS group. Q4, the highest OBS group. The model 1 was a crude model. The model 2 was adjusted for age and race/ethnicity. Model 3 was adjusted for age, race/ethnicity, educational background, poverty-to-income ratio, energy intake, hypertension, diabetes, and sleep disorder.

### 3.4 Dietary/lifestyle OBS and its association with testosterone deficiency

This study further investigated the relationship between lifestyle OBS/dietary OBS and testosterone deficiency. The multivariable analysis (Table 5) revealed that higher lifestyle OBS scores were independently associated with a 22% reduced risk of testosterone deficiency (OR, 0.78, 95% CI, 0.73 to 0.84), whereas no significant association was found for dietary OBS.

**Table 5:** Dietary/lifestyle OBS and its association with testosterone deficiency.

	Model 1	*p*-value	Model 2	*p*-value	Model 3	*p*-value	Model 4	*p*-value
Testosterone deficiency, OR (95% CI)								
Dietary OBS	1.00 (0.98 to 1.02)	0.798	1.00 (0.98 to 1.02)	0.953	0.99 (0.96 to 1.01)	0.324	1.00 (0.97 to 1.02)	0.726
Lifestyle OBS	0.80 (0.75 to 0.85)	<0.001	0.78 (0.72 to 0.83)	<0.001	0.78 (0.73 to 0.84)	<0.001	0.78 (0.73 to 0.84)	<0.001

OR, odds ratio; CI, confidence interval; OBS, oxidative balance score. The model 1 was a crude model. The model 2 was adjusted for age and race/ethnicity. The model 3 was adjusted for age, race/ethnicity, educational background, poverty-to-income ratio, energy intake, hypertension, diabetes, and sleep disorder. The model 4 was adjusted for dietary OBS or lifestyle OBS based on model 3 additionally.

### 3.5 Additional analyses

**Figure 3:**
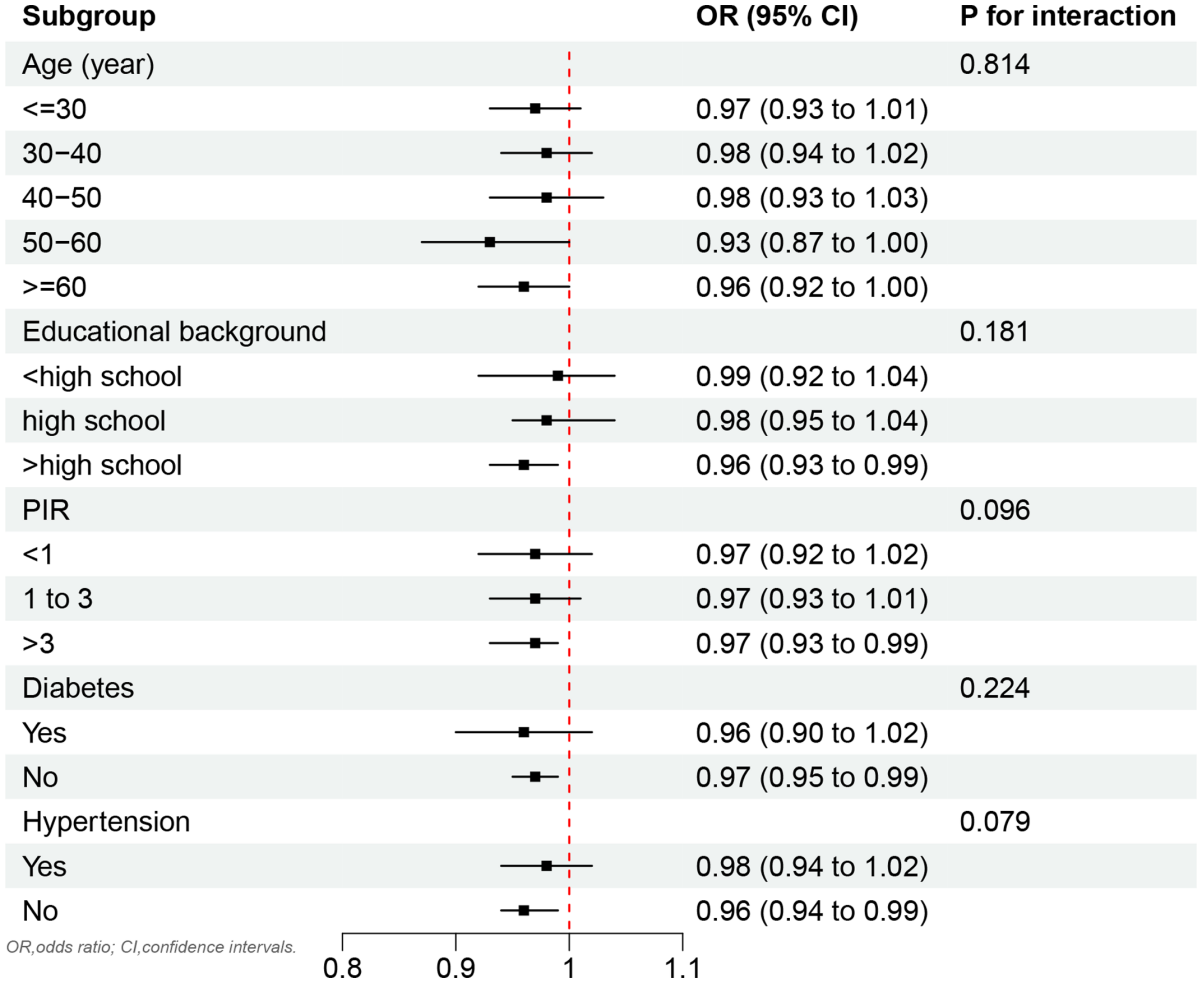
Subgroup analyses by age, educational background, PIR, diabetes, and hypertension.

As shown in Figure 3, no significant association was observed between OBS and testosterone deficiency across the stratified age groups. However, a significant inverse correlation between OBS and testosterone deficiency was identified in subgroups with an educational background above high school, as well as in individuals without hypertension and diabetes.

Importantly, the relationship between OBS and testosterone deficiency remained highly robust, even after the sequential removal of each OBS component (Supplementary material S1).

## 4 Discussion

In this study, which utilized a nationally representative sample of the U. S. population, we primarily identified an inverse association between OBS and testosterone deficiency in males. The highest OBS group exhibited a 38% reduction in the risk of testosterone deficiency compared to the lowest OBS group. A significant inverse correlation was observed between lifestyle OBS and testosterone deficiency, whereas no such association was found for dietary OBS. Furthermore, factors such as educational level, socioeconomic status, and people without chronic conditions (e.g., hypertension and diabetes) appeared to influence this relationship.

Research on the relationship between diet and disease has consistently been a prominent area of interest, and this holds true for studies focusing on testosterone deficiency as well. Although the dietary OBS alone did not demonstrate a statistically significant association with testosterone deficiency in males, the composite OBS showed a robust inverse correlation. This discrepancy warrants thorough discussion. The Mediterranean diet, which includes a variety of antioxidant-rich components, has been shown to significantly elevate testosterone levels when adhered to over the long term (26). In men, dietary fiber intake is associated with a modest increase in testosterone levels, which can be attributed to the influence of dietary factors on hormonal metabolism (27). However, studies have also revealed that men adhering to a low-fat diet tend to exhibit lower testosterone levels (28). Zinc, a widely recognized antioxidant, plays a critical role in testicular metabolism, and its deficiency may consequently affect testosterone levels (29). Basic research has also demonstrated that the combined application of zinc and selenium enhances testosterone levels by strengthening antioxidant mechanisms (30). Nicotinic acid intake has been shown to reverse the decline in testosterone levels caused by testicular damage, demonstrating a positive effect on improving testicular function (31). Surprisingly, a high-fat diet in parental generations can lead to the occurrence of testosterone deficiency in offspring (32). However, a study has also shown no association between cholesterol intake and testosterone levels (17). From these research findings, it is evident that most studies have focused on the impact of a single dietary modification on testosterone deficiency, without considering multiple or overall dietary patterns. Therefore, this study, which integrates numerous dietary factors to investigate their relationship with testosterone deficiency, is well-justified, and its results are likely to be more reliable. Consequently, an integrated assessment incorporating both dietary and lifestyle factors is essential for advancing our understanding of disease pathogenesis. A comprehensive, multidimensional approach represents a fundamental prerequisite for elucidating disease mechanisms.

Many lifestyle habits can also significantly influence testosterone levels. Long-term alcohol consumption can impair the male gonadal axis and lead to a reduction in testosterone levels (33). Cotinine, a metabolite of cigarette smoke, exhibits a non-linear relationship with testosterone levels. Once cotinine levels in the body reach a certain threshold, they are negatively correlated with testosterone (34). However, previous studies have also indicated that smokers tend to have higher testosterone levels compared to non-smokers (35). Moderate physical activities can increase testosterone levels, thereby maintaining male physiological functions and combating aging (36). In males, there exists an inverse correlation between obesity and testosterone levels (37). Fat accumulation due to obesity leads to increased oxidative stress levels in males (38). The BMI levels are positively correlated with testosterone deficiency, suggesting that addressing obesity may offer a feasible approach to mitigating this condition (39). In this study, it was also demonstrated that lifestyle OBS is inversely correlated with testosterone deficiency. Promoting a healthy lifestyle is therefore essential, as modifying lifestyle habits appears to be a viable approach. However, further prospective studies are needed to validate these findings.

Social factors are closely associated with the development of diseases. Patients with private insurance can utilize telemedicine for the diagnosis and treatment of testosterone deficiency, indicating that individuals require a certain level of financial resources and capability (40). Males with testosterone deficiency who struggle with adverse socioeconomic conditions appear less likely to seek or adhere to formal treatment regimens (41). Furthermore, males with type 2 diabetes often exhibit testosterone deficiency, which provides new insights into the prevention of testosterone deficiency in non-diabetic individuals (42). Particularly in obese populations, educational level and hypertension serve as significant mediators between obesity and testosterone deficiency (43). Therefore, the findings of this study also highlight the importance of identifying social heterogeneity and integrating male lifestyle factors to develop appropriate health policies.

This study revealed the relationship between OBS and testosterone deficiency, offering several strengths. First, the participants represent a diverse and nationally representative sample of the U. S. population, accurately reflecting its demographic variability. Second, the analysis incorporated weighted data processing, ensuring the representativeness and robustness of the results. Third, the study utilized high-quality data from a publicly accessible database that provides comprehensive health and nutritional information. However, the limitations of this study must also be acknowledged. First, the primary limitation is that the study relies on cross-sectional data, making it difficult to infer causal relationships. Second, some data were based on participant recall, which introduces potential bias. However, the large sample size of this study may help mitigate this issue. Third, the exclusion of participants with significant missing data led to substantial sample attrition, though the results remain relatively robust and realistic. In addition, prospective studies incorporating computer-aided sperm analysis parameters are needed to fully elucidate these relationships.

In addition, although we performed a sensitivity analysis by removing individual OBS components in sequence, we acknowledge that this method may have limited ability to assess the robustness of the association, especially given the lack of an independent relationship between Dietary OBS and testosterone deficiency. This approach offers preliminary insight but may not fully reflect the complex interplay between lifestyle and dietary contributors to oxidative stress. Future studies should explore alternative methods, such as weighted composite scores or interaction models, to better delineate the independent and joint effects of OBS components. The study adjusted for potential confounding factors, the study only showed that OBS was associated with testosterone deficiency, however, higher OBS may represent individuals with other healthier lifestyles, which may also affect testosterone levels. Future studies in this domain are warranted to further elucidate these mechanisms.

## 5 Conclusion

In summary, the study demonstrated that higher OBS, particularly the lifestyle OBS, was significantly associated with reduced risk of testosterone deficiency in males. These findings highlight the crucial role of dietary and lifestyle antioxidants in male testosterone deficiency. However, further multicenter, large-scale prospective studies are required to validate these findings. In conclusion, this study offers a novel perspective for the treatment and management of male testosterone deficiency, leveraging this relationship.

## Data Availability

Publicly available datasets were analyzed in this study. This data can be found at: https://wwwn.cdc.gov/nchs/nhanes/.
