# Supplementary material for: Association between the oxidative balance score and testosterone deficiency in males: a cross-sectional study
Source: Front Nutr. 2025 Jul 23;12:1577823. doi: 10.3389/fnut.2025.1577823 (PMC12325012; doi:10.3389/fnut.2025.1577823)
Supplement: Supplementary file 1 [file Table_1.docx]

Supplementary Material

# Association between the oxidative balance score and testosterone deficiency in males: A cross-sectional study

Ze Zhuge^1^, Kaihui Zheng^1^, Xiaojun Ji^1^, Xiaobo Wang^1^, Xuena Zhang^2*^

^1^ Wenzhou Central Hospital, Wenzhou, China.

*** Correspondence:**Corresponding Author: Xuena Zhang, zhangxuena29@163.com

# Supplementary S1.

# Sensitivity analyses to assess the effects of individual Oxidative balance score components on the Testosterone deficiency in NHANES 2011-2016

| OBS | Testosterone deficiency | |  |
| --- | --- | --- | --- |
| OBS original model 3 | OR (95% CI) | *P*-value |  |
| OBS excluding dietary fiber | 0.97 (0.94 to 0.99) | 0.008 |  |
| OBS excluding carotene | 0.97 (0.94 to 0.99) | 0.008 |  |
| OBS excluding riboflavin | 0.96 (0.94 to 0.99) | 0.006 |  |
| OBS excluding niacin | 0.97 (0.94 to 0.99) | 0.010 |  |
| OBS excluding vitamin B6 | 0.96 (0.94 to 0.99) | 0.007 |  |
| OBS excluding total folate | 0.97 (0.94 to 0.99) | 0.006 |  |
| OBS excluding vitamin B12 | 0.96 (0.94 to 0.99) | 0.005 |  |
| OBS excluding vitamin C | 0.97 (0.94 to 0.99) | 0.006 |  |
| OBS excluding vitamin E | 0.97 (0.94 to 0.99) | 0.007 |  |
| OBS excluding calcium | 0.96 (0.94 to 0.99) | 0.008 |  |
| OBS excluding magnesium | 0.96 (0.94 to 0.99) | 0.006 |  |
| OBS excluding zinc | 0.96 (0.94 to 0.99) | 0.004 |  |
| OBS excluding copper | 0.96 (0.94 to 0.99) | 0.005 |  |
| OBS excluding selenium | 0.97 (0.94 to 0.99) | 0.006 |  |
| OBS excluding total fat | 0.97 (0.95 to 0.99) | 0.008 |  |
| OBS excluding iron | 0.97 (0.95 to 0.99) | 0.012 |  |
| OBS excluding physical activity | 0.98 (0.96 to 0.9998) | 0.047 |  |
| OBS excluding alcohol | 0.97 (0.94 to 0.99) | 0.007 |  |
| OBS excluding body mass index | 0.98 (0.96 to 1.01) | 0.176 |  |
| OBS excluding cotinine | 0.96 (0.94 to 0.99) | 0.005 |  |
| OR, odds ratio; CI, confidence intervals. OBS, oxidative balance score.  The model 3 was adjusted for, age, race/ethnicity, educational background, poverty-to-income ratio, energy intake, hypertension, diabetes, and sleep disorder. | | |  |
|  |  |  |  |
|  |  |  |  |
|  |  |  |  |
|  |  |  |  |
